# The AD Knowledge Portal: an open‐access platform for sharing data, tools, and results of aging and dementia research

**DOI:** 10.1002/alz70855_106126

**Published:** 2025-12-24

**Authors:** Jo Scanlan, Trisha M Zintel, Zoë Leanza, Jessica S Britton, Amelia Kallaher, Jaclyn Beck, Anthony Peña, William L. Poehlman, Jesse C Wiley, Victor Baham, Melissa Klein, Abby Vander Linden, Anna Greenwood, Karina Leal, Jessica Malenfant, Laura M Heath, Susheel Varma

**Affiliations:** ^1^ Sage Bionetworks, Seattle, WA, USA

## Abstract

**Background:**

The AD Knowledge Portal (https://adknowledgeportal.org) is a National Institute on Aging (NIA) sponsored resource for Alzheimer's Disease data and other research tools.

**Method:**

The AD Portal allows researchers to share human and non‐human data with appropriate attribution for reuse, and in compliance with necessary data governance and ethical guardrails. This is accomplished through the use of the secure Synapse data sharing platform and a user‐friendly data portal website.

**Results:**

The AD Portal hosts resources from 14 NIH‐funded research programs and 97 grants related to dementia and aging, including ∼800TB of data from over 11,000 individuals. This diverse collection includes next‐generation sequencing, imaging, and behavioral data from brain banks, longitudinal cohorts, cell lines, and animal model systems. A decade after its inception, the Portal remains an important resource for contemporary AD research, as demonstrated by the ∼290TB of multimodal single‐cell omics data currently available.

Users can also explore experimental mouse and marmoset models, publications, and summarized evidence for putative AD drug targets via multiple integrated results explorers. Portal data can be combined with external research datasets via interoperability with cloud‐based analysis platforms including CAVATICA and Terra (in development). These integrations provide users with an alternative to direct download and enable data reuse.

In total, 12.57 PB of data from the Portal has been downloaded by more than 6,000 unique users since January 2022. Average downloads (TB/month) increased by 79% year‐over‐year between 2022 and 2023 and doubled between 2023 and 2024. Expansion of available resources and increases in both volume and number of data downloads correlates with a similar increase in data reuse. Portal data has been referenced in over one thousand publications since 2019, with more than half of those publications representing secondary data reuse.

**Conclusion:**

The AD Knowledge Portal continues to serve the research community by sharing rich, high‐throughput data and other resources that enable novel discoveries in the field of Alzheimer's disease. Upcoming features include interoperability with AD Workbench, NACC, NIAGADS, and LONI and valuable new data types, including spatial transcriptomics and longitudinal molecular and behavioral data from mouse and marmoset models of AD.

